# Pulmonary Arterial Hypertension in Hispanics

**DOI:** 10.7759/cureus.5834

**Published:** 2019-10-04

**Authors:** Raiko Diaz, Gustavo Ferrer

**Affiliations:** 1 Pulmonary Medicine, Aventura Hospital and Medical Center, Aventura, USA; 2 Pulmonary and Critial Care Medicine, Aventura Hospital and Medical Center, Aventura, USA

**Keywords:** pulmonary hypertension, pulmonary medicine, hispanic/latino health

## Abstract

Pulmonary hypertension (PH) is a medical condition characterized by elevated pressures in the pulmonary vessels. Pulmonary arterial hypertension (PAH), or pre-capillary PH, is a subgroup of the broader PH definition. PAH is rare compared to other groups of PH; its prevalence is about 15 cases per million in the adult population. Several disease processes may lead to PAH; however, the most common cause of PAH is idiopathic. Until recently, treatment for PAH was very limited and prognosis was dismal. Limitations in management remain present today but more treatment options are available for patients suffering from this condition.

Most of the information available regarding PAH comes from registries in the United States and Europe. Limited information about epidemiology, treatment options, and response to the treatment is available for other ethnic groups such as Hispanics. In the world of medicine, we have learned from other more common medical conditions that components, such as genetics, environment, and culture may affect how diseases manifest and how they respond to treatment. It is important to be aware of how different ethnic groups exposed to different environmental conditions respond to different treatment modalities. The aim of this paper is to review the limited data available regarding PAH in Hispanics. This paper will review the information regarding the etiology, diagnosis, and treatment modalities available in South American and Central American countries. This paper will also review the data available for Hispanics diagnosed with PAH living in the United States. The goal is to highlight the difference in how PAH manifests in Hispanics compared to other ethnic groups. We aim to emphasize the importance of the lack of data available for this group and how it may be affecting the way we are treating Hispanics with PAH.

## Introduction and background

Pulmonary arterial hypertension (PAH) is a subgroup of a broader medical condition known as pulmonary hypertension (PH). PH is a disease process which is characterized by elevated pressures in the pulmonary vessels. Different etiologies may cause the elevated pressures and therefore PH is classified into five different groups based on the etiology. Group 1 is pulmonary arterial hypertension, PAH, (drugs, connective tissue disease, and even genetics may predispose individuals to PAH); Group 2 is secondary to left-sided heart disease; Group 3 is due to chronic lung disease and hypoxemia; Group 4 is due to chronic thromboembolic disease (chronic thromboembolic pulmonary hypertension, CTEPH), while Group 5 is due to unidentified mechanisms [1]. PH may also be classified into pre-capillary PH (primary elevation of the pressures in the pulmonary arterial system) or post-capillary PH (primary elevation of pressure in the pulmonary venous system) [1].

The most common symptoms associated with PH are dyspnea and fatigue on exertion; such symptoms are secondary to inadequate increase in cardiac output during exercise. Other symptoms may include exertional chest pain, exertional syncope, weight gain from edema, and even abdominal pain and swelling due to hepatic congestion. Physical examination may reveal increased intensity of the pulmonic component of the second heart sound, as well as signs of right ventricular failure in more advanced cases, such as elevation of jugular venous pressure, murmur of tricuspid regurgitation, hepatomegaly, ascites, and peripheral edema [2]. Imaging modality for initial diagnosis includes chest X-ray, which may show enlargement of the central pulmonary arteries. CT scan of the chest classically shows the main pulmonary artery enlarged compared to the ascending aorta. The ratio of the diameter of the pulmonary artery to the ascending aorta is usually greater than one in patients with PH. Ventilation perfusion imaging may be used for the evaluation of PH secondary to CTEPH. Laboratory studies for diagnosis of PH are nonspecific; however, if clinical suspicions are high, an elevated brain natriuretic peptide (BNP) may indicate evidence of right ventricular failure in advance cases of PH [2-3]. 

Once PH is suspected, diagnostic testing is used to confirm the diagnosis, assess the severity, and identify the potential etiologies. Detailed history should focus on any evidence pointing towards connective tissue disease, history of smoking or chronic lung pathology, prior use of medications such as appetite suppressants or drugs such as cocaine, risk of having HIV or liver disease, and prior history of thromboembolic disease [1]. 

Transthoracic echocardiography (TTE) is usually the initial step in diagnosing PH. Tricuspid regurgitant jet velocity may be used to estimate the pulmonary artery systolic pressure (ePASP). The right ventricle size, wall thickness, and function may be assessed, as well as the left ventricular function to assess potential contribution of left-sided heart disease. It is important to know that the ultimate diagnosis must be made from values obtained from a right heart catheterization (RHC), as studies have shown that ePASP has poor correlation with values obtained directly from RHC [3]. Other studies include pulmonary function test (PFT), high resolution CT (HRCT) scan of the chest, and 6-minute walk test (6MWT) for the evaluation of Group 3 PH. A ventilation perfusion imaging may be necessary to evaluate for CTEPH if suspecting Group 4 PH [3].

Right heart catheterization is the gold standard for the diagnosis of PH. It may be used to differentiate between the different groups of PH. It is particularly important for the diagnosis of Group 1 and Group 2 PH, as well as for suspected mixed etiology for PH. Left heart catheterization may also be useful to rule out ischemic cardiac disease. PAH, or Group 1 PH, is diagnosed when the mean pulmonary artery pressure is > 20 mmHg, the pulmonary wedge pressure (PCWP) is less than 15 mmHg, and when pulmonary vascular resistance is more than three wood units [4]. RHC may also be used to assess the response to calcium channel blockers; this is called the vasoreactivity test. Patients who respond to the vasoreactivity test may be treated with calcium channel blockers, while those who do not, will likely need PH-specific therapy with a prostanoid, endothelin receptor antagonist, phosphodiesterase 5 inhibitor, or soluble guanylate cyclase stimulant [5].

Little is known about the epidemiology of PH outside of populations in the United States and Europe. These countries have extensive registries documenting the prevalence and etiology of the disease; however, the data in Latin American countries are scarce. Information regarding genetics and epidemiology for different ethnicities and geographical locations is important as the epidemiology and treatment of the disease may differ significantly between different ethnic groups. Literature review reveals limited studies and registries in Latin American countries which may shed some light on this topic [6].

## Review

Most of the data available regarding the etiology, diagnosis, and treatment of PAH in Latin American countries come from three different registries. The information comes from registries in Argentina, Chile, and Brazil. Most of the data in these registries is for Group 1 PH, or PAH, and more specifically idiopathic PAH. The prevalence of PAH in these registries is higher than the registries available for the United States and Europe [6]. 

It is also important to recognize that there are different common etiologies for PAH even between Latin American countries. For example, in Brazil, schistosomiasis is one of the main causes of PAH (about 20% of cases), while in Argentina congenital diseases are an important cause of PAH (about 28% of cases) [7-8]. Connective tissue disease is an important cause of PAH in Brazil, almost similar in prevalence when compared to the US and European countries; however, it is not as frequently seen in Chile and Argentina [7].

Another key difference is the severity of the disease. Most patients from the Latin American registries were in NYHA/WHO functional class III or IV (around 50%) which is lower than the documented cases in registries from the United States and Europe. The 6-minute walk distance was also higher in the three Latin American databases compared to the US REVEAL registry [6].

It is important to mention that the three registries from Latin America showed the average age of diagnosis being much lower compared to registries in the US and European nations. Mean age of diagnosis varied between 34 and 51 in Latin American countries compared to over 50 in the US and European registries. All studies also showed a similar prevalence in females (around 60%-86%) to the prevalence in the US and European countries (around 70%) [6].

Curiously, in terms of survival, the Brazilian and Argentinian registries showed a slightly higher survival rate compared to US and European registries. This observation may be due to the younger age at diagnosis and lack of other comorbidities seen in these populations [7-8]. Such findings are despite the limitations when it comes to treatment modalities available in Latin American countries. Based on data from the Latin American registries, oral therapy with sildenafil is the most common treatment modality. Most combination therapies or more advanced IV therapies are either not approved for use or not widely available to patients diagnosed with PAH; therefore, data for more advanced therapies are not available in the registries [6].

Data regarding the prevalence and etiologies of PAH in Hispanics living in the United States are also limited. The NIH and the REVEAL registries in the United States provide the most information for different races/ethnicities in patients with the diagnosis of PAH. The NIH registry reports that about 2.3% of patients with the diagnosis of PAH are Hispanics [9]. The REVEAL database enrolls 72.8% white patients and 18.5% non-white, 8.9% of which are Hispanics. Both registries enroll a percentage of Hispanic patients which is lower than the expected prevalence of 11.5% [10]. The disparity likely indicates under-representation of Hispanic patients in both registries. Both registries showed that the female to male ratio was higher in the Hispanic population compared to the white population. The REVEAL registry also indicates that Hispanic patients were more likely to have congenital heart disease as the cause of PAH compared to other ethnicities. Interestingly, analysis of the data in the REVEAL registry shows that mortality in Hispanics was lower or equal to that in non-Hispanic whites [10-11].

The conclusion from all the database analysis when it comes to the prevalence of PAH among Hispanics living in the United States as well as in Latin American countries is that the data for this population are very limited. The limitations are likely due to several reasons, including the lack of data coming from most Latin American countries, the small percentage of Hispanics enrolling in clinical trials in the United States, and socioeconomics. Socioeconomics is particularly important because in many cases the lack of resources in the Hispanic population leads to delay in diagnosis as well as medical treatment [12]. Unfortunately, this has led to poor understanding with regard to pathophysiology in this population. Genetics is likely to play a role in the development of PAH and likely in the way patients respond to different therapies. It is believed that different races respond differently to hypoxemia at high altitudes. An example is the Tibetan population; the Tibetans have lived at an elevation of over 4000 m for over 25,000 years. Natural selection over the years has led to genetic changes that have allowed them to adapt to a hypoxic environment. Extensive research has been done in this population and they seem to exhibit a relative resistance to developing PH [13].

The lack of data for the Latin American and US Hispanic population possibly also has implications when it comes to treatment and management of the disease. Genetics and environmental exposures likely play a role in the efficacy of different medications in different races and populations, in a way similar to how different races respond differently to medications such as angiotensin converting enzyme inhibitor (ACEi). One PAH medication that has shown to have different efficacy rates in different races is the class of medication known as endothelin-receptor antagonists. Gabler et al. completed an analysis of six randomized controlled trials comparing outcomes of using this medication based on demographic factors. The result was that in the white population the 6MWD increased by 41.6 m while in black patients it only increased by 3.5 m. It was hypothesized that black patients have higher levels of endothelin-1, and therefore need a higher dose of the medication compared to white patients [14]. These findings emphasize the fact that most trials for PAH medications have been done in white patients and the doses are mostly targeted to that population. When it comes to trials identifying the percentage of Hispanic patients who participated, the information is even more limited. In fact, only three medical trials for PAH treatment since the year 1992 have acknowledged participation of Hispanics [12].

## Conclusions

In conclusion, it is important to raise awareness among Hispanics in Latin American countries and the United States regarding PH. Awareness of this disease is limited among these populations and this likely leads to under diagnosis as well as delayed treatment. It is also important to increase the percentage of this population participating in clinical trials and collect data based on different races. This would lead to better understanding in the interaction between environmental exposure, genetics, pharmacology, and response to treatment. 
